# Expression Change of miR-214 and miR-135 during Muscle Differentiation 

**DOI:** 10.22074/cellj.2015.7

**Published:** 2015-10-07

**Authors:** Maryam Honardoost, Masoud Soleimani, Ehsan Arefian, Mohammad reza Sarookhani

**Affiliations:** 1 Department of Molecular Medicine, School of Medicine, Qazvin University of Medical Sciences, Qazvin, Iran; 2 Cellular and Molecular Research Center, Qazvin University of Medical Sciences, Qazvin, Iran; 3 Department of Molecular Biology and Genetic Engineering, Stem Cell Technology Research Center, Tehran, Iran; 4 Department of Hematology, School of Medical Sciences, Tarbiat Modares University, Tehran, Iran; 5 Department of Microbiology, School of Biology, College of Science, University of Tehran, Tehran, Iran

**Keywords:** Myoblast, Differentiation, miR-214, miR-135

## Abstract

**Objective:**

MicroRNAs (miRNAs) are a class of small non-coding RNAs that play pivotal roles in many biological processes such as regulating skeletal muscle development where alterations in miRNA expression are reported during myogenesis. In this study, we aimed to investigate the impact of predicted miRNAs and their target genes on the myoblast to myocyte differentiation process.

**Materials and Methods:**

This experimental study was conducted on the C2C12 cell line. Using a bioinformatics approach, miR-214 and miR-135 were selected according to their targets as potential factors in myoblast to myocyte differentiation induced by 3% horse serum. Immunocytochemistry (ICC) was undertaken to confirm the differentiation process and quantitative real-time polymerase chain reaction (PCR) to determine the expression level of miRNAs and their targets.

**Results:**

During myoblast to myocyte differentiation, miR-214 was significantly down- regulated while miRNA-135, *Irs2, Akt2* and *Insr* were overexpressed during the process.

**Conclusion:**

miR-214 and miR-135 are potential regulators of myogenesis and are involved in skeletal muscle development through regulating the IRS/PI3K pathway.

## Introduction

Several global approaches have been applied in order to better understand the molecular mechanism of myogenesis. Skeletal muscle is derived from the somites, the embryonic structures in mammalians that produce differentiated muscular tissue after progressive subdivisions (1). Myoblasts (immature muscle cells) exit from cell cycle after a defined proliferation time to then become terminally differentiated myocytes (1, 2). Finding the protein network underlying skeletal muscle differentiation will lead to a better understanding of muscle biology, muscle dysfunction and pathogenesis of various muscular disorders, and may provide new approaches for therapy.

The fate of myogenic precursor cells is first determined by transcription factors Pax3/Pax7, followed by regulation of myogenic differentiation (MyoD) through the expression of myogenic regulatory factors (MRFs) in the skeletal muscle lineage (3). *MyoD* is thus considered as a marker of terminal commitment to muscle fate. Muscle-specific genes, including myosin heavy chain (*MHC*) genes, are expressed in the last phase of this multiregulated program, where mononucleated myocytes specifically fuse to each other to form multinucleated myotubes (4). Table 1 provides the most important transcription factors, growth factors and related signaling pathways involved in muscle differentiation.

This myogenic process is controlled by numerous signaling pathways (5). In recent years, the mammalian target of rapamycin (mTOR) has emerged as a key regulator of skeletal myogenesis by controlling multiple stages of myogenic differentiation through distinct mechanisms (6). mTOR is a Ser/Thr kinase that operates as an important regulator of cellular differentiation (7). Several mediators of amino acid signals have been reported to lie upstream of mTOR, including its activator phosphoinositide 3-kinase (PI3K) (8).

**Fig.1:**
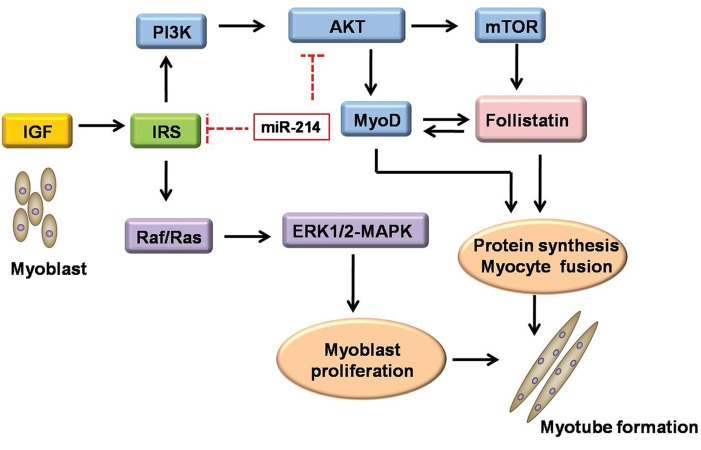
Myogenesis through the IGF/AKT/mTOR pathway. IGF is thought to initiate an autocrine signaling cascade through the IGF-I receptor, and IRS 1 and 2 that activate PI3K and mitogen-activated protein kinases (MAPK). PI3K activates AKT, however, either PI3K or AKT is sufficient for myoblast differentiation and fusion. PI3K and AKT drive differentiation by increasing the transcriptional activity of MyoD and activating the mTOR pathway. mTOR expression and activity increases during differentiation leading to an increase in the activity of its downstream target, follistatin, which prevents myostatin (the most powerful inhibitor of muscle growth) from executing its inhibitory effect on muscle development. AKT; RAC-beta serine/threonine-protein kinase, ERK1/2; Extracellular-signal-regulated kinases1/2, IGF; Insulin-like growth factor, IRS; Insulin receptor substrates, mTOR; Mammalian target of rapamycin, MyoD; Myogenic differentiation, PI3K; Phosphoinositide 3-kinase, Raf; Rapidly accelerated fibrosarcoma and Ras; Rat sarcoma.

mTOR signaling regulates myoblast differentiation by controlling the myogenic expression of insulin-like growth factor (IGF) at the transcriptional level via a muscle-specific enhancer (9). IGFs are critically involved in skeletal muscle development, adult muscle regeneration and hypertrophy (10). In cultured myoblasts, growth factor deprivation initiates the differentiation process, owing to the induction of IGF (11) and subsequent activation of the IGFreceptor (Fig .1). It then initiates an autocrine signaling cascade through insulin receptor substrates (IRS) 1 and 2 that activate PI3K and in turn a major downstream pathway mediated by and RAC-beta serine/threonine-protein kinase (AKT) (12). The IGF-1/AKT/mTOR pathway is therefore an important regulatory component which controls muscle development (13). Upon binding to its membrane receptor (IGFR1), IGF- 1 activates both extracellular-signal-regulated kinases1/2 (ERK1/2) and PI3K/AKT/mTOR pathways. ERK1/2 is required for myoblast proliferation (14), while the PI3K/Akt/mTOR pathway promotes protein synthesis and is essential for myotube formation through a MyoD/ follistatin pathway, which requires mTOR kinase activity (13, 15). Thus, PI3K/AKT pathway is a vital intracellular signaling mechanism that pivotal for muscle development (16).

The process of myogenesis is extremely complex and requires a specific organization of signaling molecules that regulate the expression of particular genes and miRNAs (17, 18).

Recently, microRNAs (miRNAs), a class of evolutionarily conserved and small non-coding RNAs (19), have emerged as novel and essential regulators of myogenesis (20). Mature miRNAs are 21– 25 nucleotides in length and are partially complementary to one or more mRNA molecules. Their main function is to down-regulate gene expression in a variety of manners including translational repression, mRNA cleavage and de adenylation (13, 21). Regulation of myogenic gene expression by miRNAs has emerged as a new level of control for myogenesis. For example, miRNAs can promote differentiation by repressing negative regulators of transcriptional activity or suppress it by repressing positive regulators. Muscle-specific miRNAs such as miR-1, miR-133, and miR-206 have a central role in myogenesis (22). Other miRNAs have also been implicated in muscle development, including miR-26a (23), miR-27b (24), miR-29 (25), miR- 125b (26), miR-155 (27), miR-128a (28) and miR- 181 (29).

Additional miRNAs have been reported to participate in skeletal myogenesis and include miR-24 (30), miR-378 (31), miR221/222 (32), miR-486 (33), miR-208b/miR-499 (34) and miR-214 (35). Furthermore, several miRNAs have been demonstrated to regulate the PI3K/AKT/mTOR pathway during myogenesis (33). Although an increasing number of miRNAs are found to function in myognenesis, knowledge about the role of individual miRNAs in the molecular network of muscle development remains poorly understood and still mainly unknown. As a new class of regulators of skeletal myogenesis, miRNAs hold the potential to identifying novel biomarkers and developing therapeutic strategies for muscular diseases. The aim of this study was to quantify expression changes of bioinformatically selected miR-214 and miR-135 and their targets, Insulin receptor substrates (*Irs2*), RAC-beta serine/threonine-protein kinase (Akt2) and insulin receptor (*Insr*) to better understand the role of miRNAs during the muscle differentiation process.

In our survey, by using the C2C12 myoblast cell line, we identified two miRNA which had significant expression change during C2C12 differentiation process.

**Table 1:** Muscle-specific factors during myogenic process


	Proliferation	Differentiation

Myogenic factors	PAX3/7, HDAC4, MEF2, IGFI	MRF (MyoD, MRF4, Myf5), MEF2, IGFII, Myogenin (MyoG), MHC, M-CK
Signaling pathway	MEK/ERK pathway	mTOR/PI3K/AKT pathway


Transition from proliferation to differentiation, which is accompanied by the down-regulation of Pax-7 and up-regulation of Myogenin and MRF-4 is dependent on both *MyoD* and the mTOR/PI3K/AKT pathway. MyoD and Myf5 are both considered markers of terminal com- mitment to muscle fate. Muscle-specific genes such as myosin heavy chain genes (*MHC* genes) and muscle creatine kinase (M-CK) are expressed in the last phase of this multi-regulated program. AKT; RAC-beta serine/threonine-protein kinase, ERK; Extracellular-signal-regulated kinases, IGF; Insulin-like growth factor, IRS; Insulin receptor substrates, MEF2; Myocyte enhancer factor2, MEK; Mitogen/extracellular signal-regulated Kinase, MRF; Myogenic regulatory factors, mTOR; Mammalian target of rapamycin, MyoD; Myogenic differentiation, HDAC4; Histone deacetylase 4 and PI3K; Phospho- inositide 3-kinase.

## Materials and Methods

### Cell culture

463 In our experimental study, C2C12 myoblast cell line was obtained from a cell bank (Stem Cells Technology Research Center, Tehran, Iran). These cells were cultured in growth medium [GM, Dulbecco’s Modified Eagle Medium, (Gibco, UK)] containing 10% fetal bovin serum (Gibco, UK) 24 hours before being induced to differentiate, at 37˚C and 5% CO_2_. 

When cell density reached 70%, cells were digested with 0.25% trypsin (Gibco, UK) and then seeded into culture dishes. When inducing C2C12 myoblasts to differentiate, cell density must reach >90% prior to changing GM to differentiation medium [DM, Dulbecco’s Modified Eagle Medium supplemented with 3% horse serum (Gibco, UK)]. The cells were subsequently incubated with DM for another 72 hours to undergo differentiation. The control cell line (the undifferentiated C2C12 line) was cultured only in growth medium for the same time period. All cell cultures were performed at least in triplicate. 

### Immunocytochemistry (ICC)

After inducing myogenic differentiation, C2C12 cells cultured in 12-well plates were then washed with phosphate-buffered saline (PBS) and fixed with 4% paraformaldehyde (Sigma, USA) for 15 minutes. Triton-100 (0.5%, Sigma, USA) was used for permeabilization. The cells were then blocked in 2% goat serum (diluted in PBS, Sigma, USA). After blocking, the cells were incubated with antiPax7 or anti-myosin primary antibody depending on cell type (Sigma, USA) at 37˚C for 1 hour and then with the secondary fluorescent antibodies (Ray Biothech, USA) at 37˚C for 1 hour. The nuclei were stained with 4΄, 6-diamidino-2-phenylindole (DAPI, Invitrogen, USA) for 30 seconds. 

### Bioinformatics-based microRNA selection

Using Target Scan 6.2 (36), miRWalk (37) and RNAhybrid (38), we generated a list of miR-214 and miR-135 candidate target genes, containing a seed site for these two miRNAs. We merely chose target genes for each miRNA which were predicted with at least two algorithms. 

### RNA isolation and quantitative real-time polymerase chain reaction (PCR)

Cells were lysed and total RNA was extracted using TRIzol (Invitrogen, USA) according to the manufacturer’s instructions. The RNA quality and concentration were estimated using denatured gel electrophoresis and spectrophotometry respectively. About 500 ng of the total RNA was reverse transcribed into cDNA using a reverse transcription kit (Fermentas, USA) with random hexamers for target genes. cDNA synthesis of miRNAs was undertaken using the Reverse Transcription System Kit (Promega, USA) with miR-specific stem-loop primers (Table 2). Quantitative real-time polymerase chain reaction (PCR) was performed in triplicate using a 40 cycle PCR in Rotor-gene Q real-time analyzer (Corbett, Sydney, Australia). Each real-time PCR reaction contained 5 μl of 2×SYBR Premix Dimer EraserTM (TaKaRa, Japan), 3 pmol of forward and reverse primers respectively, 1 μl template of cDNA and dH_2_O up to the final volume of 10 μl, followed by a melting curve analysis to confirm PCR specificity. The average threshold cycle was used for data analysis by Rotor-gene Q software (Corbett, Sydney, Australia). Gene expression levels were normalized against the expression of β-actin and Snord 47(U47) as internal controls for miRNA expression. The 2 ^−ΔΔCt^method was employed to estimate the relative expression level of each gene. All reactions were run in triplicate. 

### Comparison of real time-PCR result by a highthroughput method data

We next assessed whether the candidate gene expression levels obtained by real time-PCR are comparable with other methods. To this end, real time-PCR results were compared with microarray data available in Gene Expression Omnibus (GEO, accession #GSE4694) (39). 

### Statistical analysis

The data were presented as mean ± standard error. To determine statistical significance, Student’s t test was applied. If not indicated otherwise, the criterion for significance was set at P<0.05. 

## Results

### Bioinformatically predicted targets

**Fig.2:**
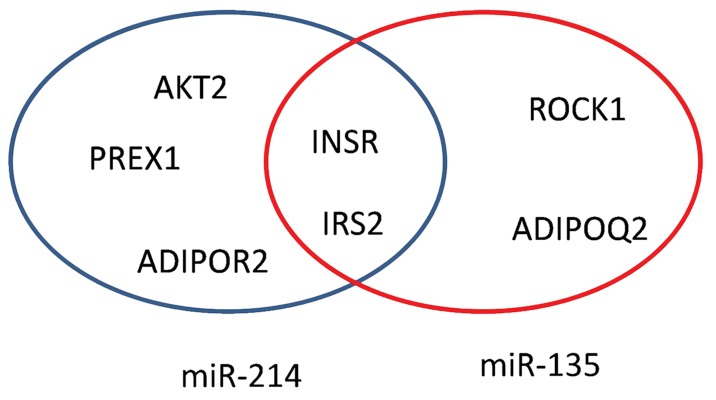
Clustering of predicted targets of miRNAs. IRS2 and INSR are mutual predicted targets of both miRNAs. ADIPOR2; Adiponectin receptor 2, ADIPOQ; Adiponectin, AKT2; RAC-beta serine/threonine-protein kinase, INSR; Insulin receptor, IRS2; Insulin receptor substrate 2, Prex1; Phosphatidylinositol 3,4,5-trisphosphate-dependent Rac exchanger 1 protein and ROCK1; Rho kinase 1.

To bioinformatically predict target genes, we initially found that miR-214 and miR-135 targeted several signal molecules regulating the myogenesis process and insulin pathway such as *IRS2, AKT2* and *INSR* (Fig .2).

**Table 2:** Gene-specific primers designed for real-time PCR assay


Primer	Sequence

Reverse transcription	
miR-214-3p-stem-loop	5ˊGTCGTATGCAGAGCAGGGTCCGAGGTATTCGCACTGCATACGACACTGC 3ˊ
miR 135a- stem-loop	5ˊGTCGTATGCAGAGCAGGGTCCGAGGTATTCGCACTGCATACGACTCACA 3ˊ
miR- 214	Forward : 5ˊGCACAGCAGGCACAGAC3ˊ
	Forward : 5ˊGCACAGCAGGCACAGAC3ˊ
miR-135a	Forward : 5ˊCGATATGGCTTTTTATTCCTA3ˊ
	Reverse : 5ˊGAGCAGGGTCCGAGGT 3ˊ
Insr	Forward : 5ˊAACAGATGCCACTAATCCTTC 3ˊ
	Reverse : 5ˊGCCCTTTGAGACAATAATCC 3ˊ
Irs2	Forward: 5ˊCAGCCAGGAGACAAGAACTC 3ˊ
	Reverse: 5ˊCGCTTCACTCTTTCACGAC 3ˊ
Akt2	Forward : 5ˊTTCGGCAAGGTCATTCTG 3ˊ
	Reverse: 5ˊTGAGGGCTGTAAGGAAGG 3ˊ


*Akt2*; RAC-beta serine/threonine-protein kinase, *Insr*; Insulin receptor, *Irs2*; Insulin receptor substrates and PCR; Polymerase chain reaction.

### Characterization of C2C12 differentiation

**Fig.3:**
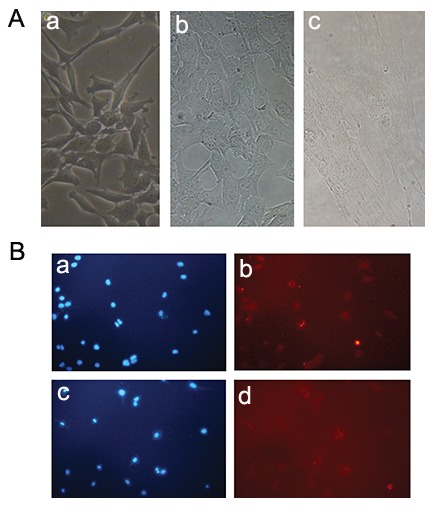
Myoblast to myocyte differentiation. A. Myoblast cells (a) differentiate into myocytes (b, c). Myocyte is indicated in part c and B. C2C12 myoblasts stained with PAX and DAPI as a positive control of precursor cells (a, b). After that, myoblasts were induced to differentiate with DMEM medium containing 3% horse serum for 3 days. The differentiated cells were seeded in a new plate and stained with MHC antibody and DAPI (c, d). DAPI; 4΄,6-diamidino-2-phenylindole, DMEM; Dulbecco’s Minimal Essential Medium and MHC; Myosine heavy chain.

Differentiation of myoblast cells to myocytes was confirmed by a positive ICC result for the specific skeletal marker, myosin .C2C12 myoblast type was confirmed by a positive ICC result for the precursor cell marker, Pax-7 (Fig .3).

### miR-214 and -135 have different expression patterns during myoblast differentiation

**Fig.4:**
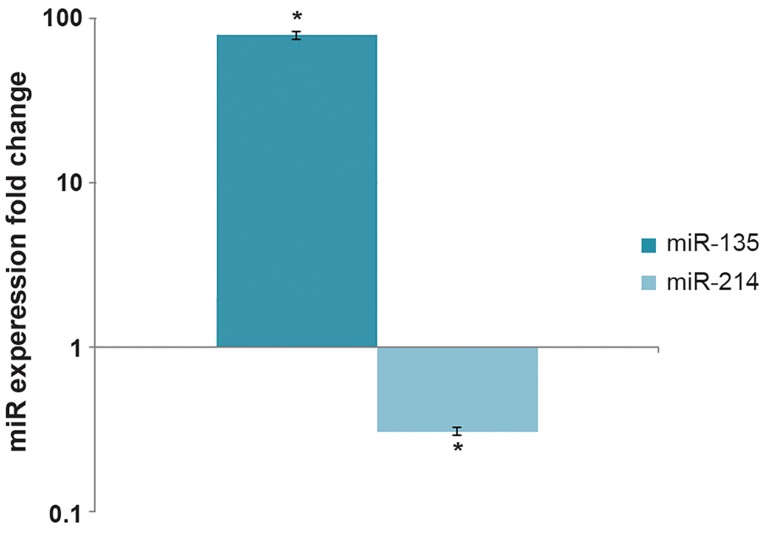
Expression pattern of candidate miRNAs during myoblast differentiation. Based on qRT-PCR results, while miR-135 was up-regulated miR-214 was down-regulated during the differentiation process.*; P≤0.05 and qRT-PCR; Quantitative real time polymerase chain reaction.

Expression profiling of miRNAs showed that miR-214 and miR-135 had significantly altered expression during myoblast differentiation with miR-214 being down-regulated and miR-135 being up-regulated more than 70-fold in differentiated cells (Fig .4).

### Changes in expression of predicted targets during C2C12 differentiation

We examined the expression of *Irs2, Akt2* and *Insr* as predicted targets of the two miRNAs studied. Interestingly, expression level of *Irs2, Akt2* and *Insr*, directly targeted by miR-214, were upregulated in differentiated cells (Fig .4).

### Comparison of real time -PCR result with microarray data

To extend the results of our quantitative real time-PCR (qRT-PCR) analysis, we analyzed all transcripts in an available microarray dataset. Chen et al. (39) had analyzed three different RNA samples from proliferating and differentiated C2C12 cells individually (6 microarrays in total).

**Fig.5:**
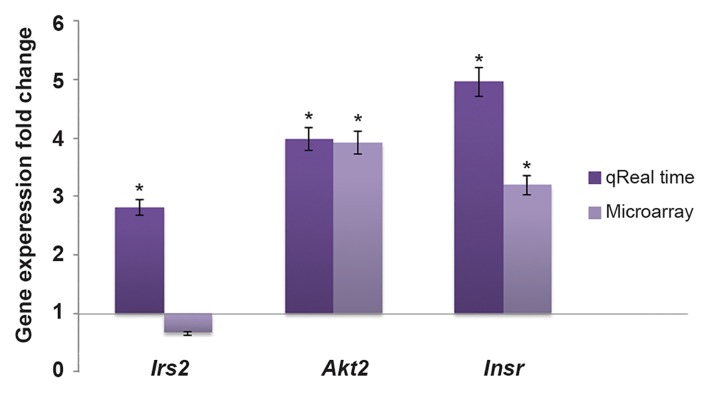
Comparison of the expression levels of predicted targets during myogenesis based on qRT-PCR and microarray analysis. The data were consistent between the two methods except for *Irs2* which was not shown to be differentially expressed in the microarray analysis (P=0.473). *Akt2*; RAC-beta serine/threonine-protein kinase, *Insr*; Insulin receptor *Irs2*; Insulin receptor substrate 2 and *; P≤0.05.

Microarray results for *Insr* and *Akt2* reflected the same up-regulation trend (P value≤0.05) but not for *Irs2* (P value=0.473, Fig .5). However, the magnitude of differential expression was different from qRT-PCR results.

## Discussion

MiRNAs play important regulatory roles in many cell processes (40,41) including the multistep differentiation process in mammalian skeletal muscle development (42). The regulation network of myogenic factors and various miRNAs is complex and appears to depend on the cell cycle and fusion stages (43). Currently, differentially expressed miRNAs are thought to be closely related to almost all aspects of muscle development and have been shown to regulate several pathways during myogenesis (43,44). 

Moreover, because of important similarities between embryonic muscle development and muscle regeneration in adults, undertaking developmental studies and particularly elucidating the roles of miRNAs in this multi-step process is valuable and may have potential clinical applications (44). In this study, we report that miR-135 was differentially expressed and may thus be involved in skeletal muscle development. Our study, for the first time, also reports that miR-135 expression was up-regulated during myogenic differentiation. miR-135 may participate in the myocyte formation process through targeting unknown components (perhaps inhibitors of muscle growth) of myogenesis in addition to those targets in our prediction (activators). On the contrary, we found that mature miR-214 was already expressed in proliferating C2C12 cells, however, it was significantly downregulated following the induction of differentiation. Furthermore, our qRT-PCR analysis showed that expression level of *Irs2, Akt2* and *Insr*, were up-regulated in differentiated cells. The upregulation of these three predicted target genes indicates that these genes are important in muscle differentiation process. 

Using an Affymetrix cDNA microarray dataset (GEO accession #GSE4694) (39), we compared the expression levels of *Irs2, Akt2* and *Insr* in undifferentiated C2C12 cells with differentiated populations. Our data were consistent with those of the microarray (P≤0.05) except for *Irs2* (P=0.473). However, qRT-PCR analysis revealed that the levels of *Insr, Irs2* and *Akt2* transcripts were shown to be more increased in comparison with microarray analysis in terms of fold change. This discrepancy is justifiable because qRT-PCR provides a more accurate representation of changes in the level of specific transcripts than the microarray analysis. This is due to the linearity of qRT-PCR results over a wide concentration range (39,45), as confirmed by serial dilution experiments with different samples (data not shown). 

Decreased expression of miR-214 accompanied with overexpression of *Irs2, Akt2* and *Insr* in C_2_C1_2_ myocytes compared with with primary muscle cells which underwent myogenic differentiation progression. 

It has been reported that muscle differentiation is blocked by decreased IRS-1/2 and PI3-K activity (3,46,47). Small RNAi-based gene silencing experiments have shown that insulin signaling pathways are dependent on IRS1/2 and are required for myotube formation and glucose uptake through the activation of AKTs (46,47). In addition, AKT promotes myoblast proliferation in cooperation with mTOR, suggesting that IRS is a key factor in inducing myoblast proliferation and myotube formation by increasing AKTs levels (47). Furthermore, AKT2 is associated with insulin signaling and the AKT/mTOR pathway which lies at the center of the regulatory network controlling muscle development (28). Gene silencing has also revealed specialized roles of AKT2 in myoblast differentiation and glucose metabolism (46). 

Interestingly, our study demonstrated that the down-regulation of miR-214 may accelerate myogenesis, because of increasing levels of its targets, *Akt2* and *Irs2*, during myogenic differentiation. Indeed, it might be the result of increasing *Irs/Akt* activation. As an important regulator of muscle development, a single miRNA can regulate the expression of many mRNA targets. Identifying the regulatory targets of miRNAs in muscle is thus crucial, however, it will be more critical to place them in a biological pathway. 

Studies in the past were largely focused on miRNAs regulating a single gene in myogenic signaling pathways (33,35). However, growing evidence suggests that miRNA can also have an effect on signal transduction pathways (13). By targeting a number of members of the same signaling pathway, miRNA can exert more profound effects than regulating one individual gene in a biological process. Thus, miR-214 down-regulation may have positive effects on myogenesis by overexpression of *Akt2, Irs2* and *Insr* in the insulin signaling pathway. 

## Conclusion

We show that miR-214 and miR-135 are potential regulators of myogenesis through regulating the IRS/AKT/PI3K pathway. Further studies such as luciferase assay and western blot and *in vivo* experience will be needed to concentrate on the complex regulatory roles of these miRNAs Understanding how miRNA regulate myogenesis will enhance our perception of the muscle development mechanism. Combining bioinformatics, biochemical and genetic approaches together will help us to elucidate the regulatory efficacy of miRNA in myogenesis and will also potentially establish new therapeutic approaches by identifying functional miRNA candidates as potential targets for clinical purposes. 
